# 1052. Characterisation of the DNA binding properties of ridinilazole, a selective antibiotic currently in phase III trials for the treatment of *Clostridioides difficile*

**DOI:** 10.1093/ofid/ofab466.1246

**Published:** 2021-12-04

**Authors:** Clive Mason, Tim Avis, Chris Coward, David Powell, Esther Duperchy, Chenlin Hu, M Jahangir Alam, Khurshida Begum, Kevin W Garey, Stefanie Reich, Stephen Moss

**Affiliations:** 1 Summit Therapeutics, Cambridge, England, United Kingdom; 2 University of Houston, Houston, Texas; 3 University of Houston College of Pharmacy, Houston, Texas; 4 Domainex, Cambridge, England, United Kingdom

## Abstract

**Background:**

*Clostridioides difficile* infection (CDI) is recognised by the CDC as an “urgent threat” in the USA, responsible for nearly 13,000 deaths, and carries an economic burden ranging from &5.4 to &6.3 billion per year. In a phase II study, ridinilazole was shown to be effective at treating CDI and decreasing subsequent recurrence compared to vancomycin. However, the precise mechanism of action of ridinilazole has yet to be fully elucidated. We now present data that reveals ridinilazole clearly co-localises with DNA in *C. difficile* and binds with high affinity to the minor groove of DNA. These interactions are predicted to have consequences on cellular functions within *C. difficile.*

**Methods:**

High resolution confocal microscopy was used to track the intracellular localisation of ridinilazole in *C. difficile.* Fluorescence intensity was used to characterise the DNA binding properties of ridinilazole; sequence specificity was demonstrated with AT- or GC-rich DNA polymers, and tight binding was shown using short double-stranded oligonucleotides. Hanging drop vapour diffusion enabled co-crystallisation and subsequent structural determination of DNA-bound ridinilazole.

**Results:**

Confocal microscopy revealed clear co-localisation of ridinilazole to the DNA within *C. difficile.* Ridinilazole demonstrated a dose-dependent increase in fluorescence in response to increasing concentration of target DNA. Fluorescence binding studies revealed that ridinilazole shows a preference towards AT-rich DNA sequences. Tight binding characteristics were demonstrated by ridinilazole in complex with short double-stranded oligonucleotides, returning dissociation constants (K_d_) of 20 – 50 nM. Crystallisation enabled co-structures of ridinilazole bound to the minor groove of double-stranded DNA oligonucleotides to be solved.

**Conclusion:**

Ridinilazole demonstrates tight binding with sequence specificity within the minor groove of DNA and co-localises with DNA in *C. difficle*. Further analysis is ongoing to fully understand this novel mechanism of action, the downstream consequences of these interactions and how they contribute to the bactericidal activity of ridinilazole.

**Disclosures:**

**Clive Mason, PhD**, **Summit Therapeutics** (Employee, Shareholder) **Tim Avis, n/a**, **Summit therapeutics** (Shareholder) **Chris Coward, PhD**, **Summit Therapeutics** (Employee, Scientific Research Study Investigator, Shareholder) **David Powell, PhD**, **Summit Therapeutics** (Employee) **Kevin W. Garey, Pharm.D., M.S., FASHP**, **Summit Therapeutics** (Research Grant or Support)

